# Low transthyretin is associated with the poor prognosis of colorectal cancer

**DOI:** 10.3389/fonc.2025.1397019

**Published:** 2025-02-05

**Authors:** Zhe Zhang, Chenhao Hu, Feiyu Shi, Lei Zhang, Ya Wang, Yujie Zhang, Xiaojiang Zhang, Junjun She

**Affiliations:** ^1^ Department of General Surgery, The First Affiliated Hospital of Xi'an Jiaotong University, Xi’an, Shaanxi, China; ^2^ Center for Gut Microbiome Research, Med-X Institute, The First Affiliated Hospital of Xi’an Jiaotong University, Xi’an, Shaanxi, China; ^3^ Department of High Talent, The First Affiliated Hospital of Xi’an Jiaotong University, Xi’an, Shaanxi, China

**Keywords:** transthyretin, prognosis, colorectal cancer, clinicopathologic feature, cancer-specific survival, nomogram

## Abstract

**Objective:**

To determine whether transthyretin (TTR) influences the prognosis of patients with colorectal cancers and establish a predictive model based on TTR.

**Methods:**

Between January 2013 and February 2019, the clinical data of 1322 CRC patients aged from 18 years to 80 years who underwent surgical treatment were retrospectively analyzed. The preoperative TTR level, clinicopathological data, and follow-up data were recorded. The X-tile program was used to determine the optimal cut-off value. Cox proportional hazard regression analysis was conducted to evaluate the correlation between the TTR and the cumulative incidence of cancer-specific survival (CSS). Nomograms were then developed to predict CSS. Furthermore, an additional cohort of 377 CRC patients enrolled between January 2014 and December 2015 was included as an external validation.

**Results:**

Based on the optimal cut-off value of 121.3 mg/L, we divided the patients into the TTR-lower group (<121.3 mg/L) and the TTR-higher group (≥121.3 mg/L). Comparative analysis revealed that the TTR-higher group exhibited a younger demographic, a higher prevalence of low colorectal cancers, an elevated R0 resection rate, superior differentiation, earlier stage and lower levels of carcinoembryonic antigen (CEA) in contrast to the TTR-lower group. The Cox multivariable analysis underscored the significance of TTR and various clinicopathological factors, encompassing age, tumor location, R0 resection status, differentiation grade, disease stage, postoperative chemoradiotherapy, and preoperative CEA levels, as substantial prognostic indicators. The postoperative survival nomogram, when internally and externally assessed, demonstrated commendable performance across multiple metrics, including the area under the receiver operating characteristic curve (AUC), calibration plots, and decision curve analysis (DCA). Compared with other models, the proportional hazards model combined with TTR demonstrates superior performance in terms of C-index, AUC, calibration chart, and DCA within the prognostic column chart.

**Conclusions:**

The preoperative TTR was identified as a prognostic factor for predicting the long-term prognosis of CRC patients who underwent surgical treatment, supporting its role as a prognostic biomarker in clinical practice.

## 1 Introduction

Colorectal cancer (CRC) has risen to become the third most common cancer globally, with the second-highest mortality rate among all cancers. In 2020, over 1.8 million cases of colorectal cancer were reported, resulting in nearly 1 million mortalities, constituting approximately one-tenth of all cancer cases and fatalities (1). Increased exposure to tobacco use, unhealthy lifestyle, alcohol consumption, stress, and overweight has contributed to the rapid surge in CRC incidence and mortality rates (2). Notably, from 1990 to 2019, a significant upsurge in incidence and mortality rates has been observed among individuals under 50 years old, particularly in countries with a high sociodemographic index. This trend imposes a substantial financial burden on society (3). Despite the widespread adoption of the tumor-node-metastasis (TNM) staging system for evaluating CRC prognosis (4), advancements in clinical research and proteomics have unveiled multiple factors—such as R0 resection, pathology, immunity, metabolism, and the tumor microenvironment—significantly impacting prognosis (5–9). Moreover, extensive research has indicated that several serological indicators and plasma markers hold potential for predicting the prognosis of CRC patients (10–14). The quest for novel markers to assess the status of CRC patients holds paramount importance in determining survival and prognosis (15).

Transthyretin (TTR) is an extracellular protein and is mainly synthesized in the liver, choroid plexus, and retinal pigment epithelium (16). It transports the thyroid hormone thyroxine and the retinol-binding protein bound to retinol (17). Some cardiologists and neurologists have reported that TTR is also associated with cardiac amyloidosis and familial amyloid polyneuropathy (18–20). Also, TTR has been used as an indicator to assess the nutritional status along with serum albumin, body mass index (BMI), and prognostic nutritional index (PNI), especially in patients with terminal cancer or patients in severe conditions (21–24). Various levels of TTR have been detected in screening trial specimens among prostate cancer, lung cancer, and ovarian cancer, which indicates TTR could be a potential marker of malignant tumors (25). Some researchers have confirmed that lower serum TTR levels may be a factor in the poor prognosis of gastric cancers, hepatocellular carcinoma, intrahepatic cholangiocarcinoma, and non-small cell lung cancer (26–28). The up-regulated transthyretin can improve the chemosensitivity of ovarian cancer, thereby improving the prognosis and survival of patients (29).

However, few literatures have reported the interaction between TTR and CRC, and whether TTR can influence the prognosis of patients who were diagnosed with colorectal cancer. Additionally, there has been no research to establish a prognostic survival prediction model based on TTR. Considering its key role in physiology and clinical nutriology, we believe that TTR might affect the prognosis of colorectal cancer.

To confirm the assumption, we examined the relationship between TTR and clinical outcomes through a retrospective case-control study and established a prognosis model. This research aimed to evaluate the clinical predictive value of TTR for cancer-specific survival (CSS) in CRC patients undergoing surgical treatment. As far as we know, this research is the first to evaluate the importance of TTR in the prognosis of colorectal cancer patients and establish a predictive model.

## 2 Materials and methods

### 2.1 Patients

**Figure 1:**
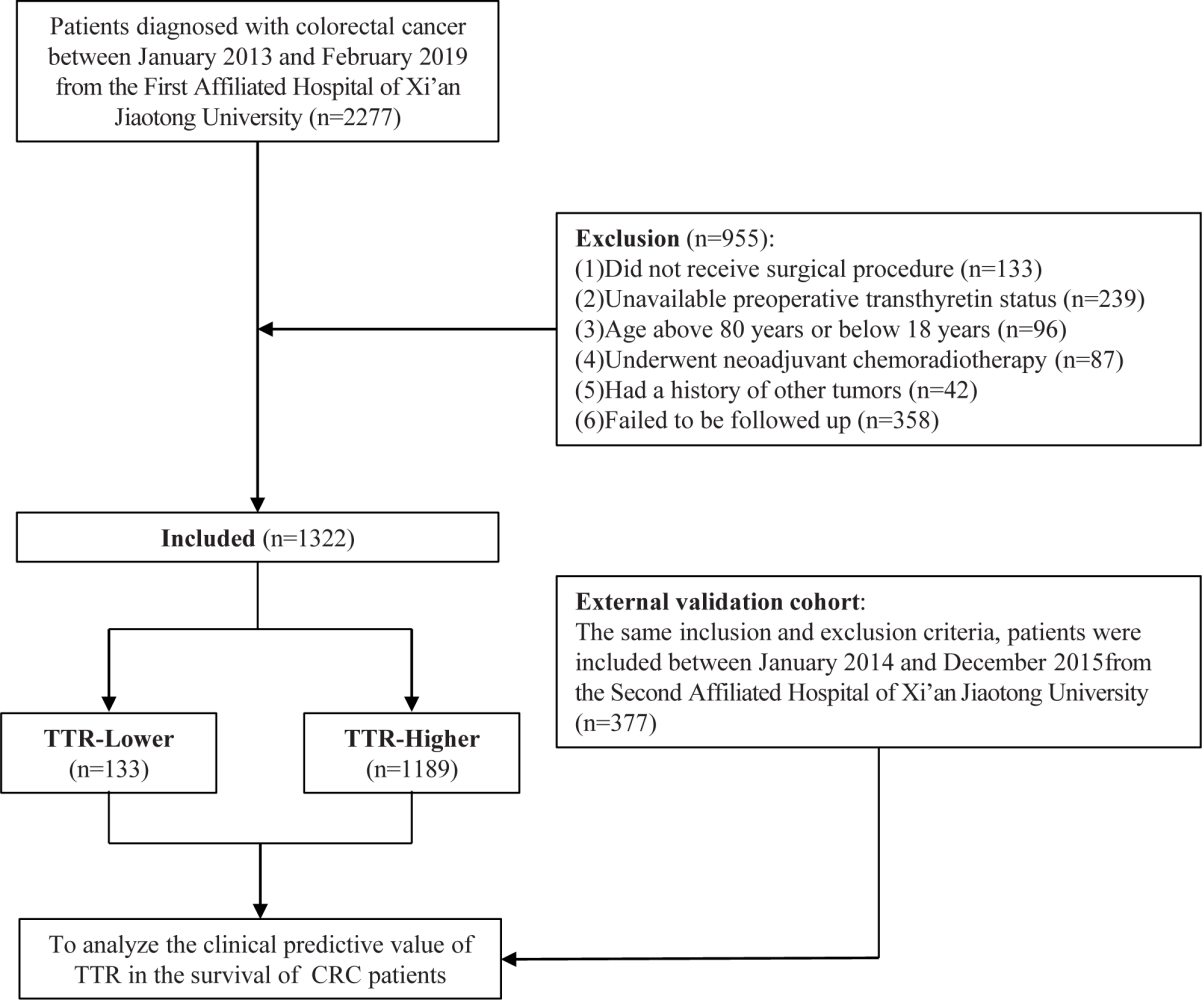
The flowchart of the study.

This retrospective case-control study enrolled 2277 consecutive patients diagnosed with colorectal cancer who received surgical resection at First Affiliated Hospital of Xi ‘an Jiaotong University between January 2013 and February 2019. The exclusion criteria and study flow chart are presented in **Figure 1**. Therefore, a total of 1322 patients diagnosed with colorectal cancer were finally included in this study as the training cohort. Additionally, 377 patients from the Second Affiliated Hospital of Xi’an Jiaotong University, diagnosed between January 2014 and December 2015, were included with the same inclusion and exclusion criteria to form the external validation cohort. The study adhered to the principles outlined in the Declaration of Helsinki. All patients provided written informed consent for data collection and subsequent analyses. The Institutional Review Board and Ethical Committee of the First Affiliated Hospital of Xi’an Jiaotong University approved this study (XJTU1AF2019LSK−227).

### 2.2 Preoperative evaluation and diagnosis

Upon admission, patients underwent a comprehensive evaluation, including imaging studies and preoperative assessments such as serological tests. Liver biochemical parameters and TTR levels were measured using the Beckman Coulter automated biochemical analysis system, performed by the Clinical Laboratory Department of our hospital. Based on the preoperative findings, an initial diagnosis was established.

### 2.3 Treatment for colorectal cancer

For patients with colon cancer clinically staged as cT_1-4_, N_0-2_, M_0_ (stages I-III), laparoscopic or robot-assisted colectomy with regional lymph node dissection is recommended. If postoperative pathology confirms negative surgical margins, the procedure is defined as an R0 resection. Among these patients, those classified as non-low risk or stage II and above are recommended to undergo adjuvant chemotherapy. Chemotherapy regimens include single-agent chemotherapy (oral capecitabine or biweekly intravenous administration of 5-FU/LV) and combination chemotherapy. Combination regimens consist of CAPEOX (XELOX), which includes oxaliplatin and capecitabine, or mFOLFOX6, which includes oxaliplatin, leucovorin (LV), and 5-FU. For patients with colon cancer clinically staged as T_4b_M_0_, palliative surgery is performed, which is classified as non-R0 resection. Postoperative treatment includes single-agent chemotherapy with 5-FU or combination chemotherapy. For patients with T_4b_ sigmoid colon cancer, local radiotherapy may also be employed. For metastatic colon cancer with resectable liver metastases, both colectomy and liver metastasectomy are performed. If postoperative pathology confirms negative margins, the procedure is defined as R0 resection. Postoperative chemotherapy regimens include single-agent chemotherapy or combination regimens. For metastatic colon cancer deemed unresectable but complicated by symptoms like bleeding, perforation, or obstruction, palliative surgery is performed and classified as non-R0 resection. Postoperative chemoradiotherapy is recommended for these patients.

For rectal cancer patients with a preoperative clinical stage of cT_1-2_N_0_, we recommend laparoscopic or robot-assisted radical rectal resection. If postoperative pathology confirms negative surgical margins, it is defined as an R0 resection. For patients with a strong desire to preserve the anus, postoperative concurrent chemoradiotherapy is advised. Postoperative radiotherapy parameters are set at 50-54 Gy over 25-30 fractions. Postoperative chemotherapy regimens include single-agent chemotherapy, as well as combination regimens such as CAPEOX (XELOX) and mFOLFOX6. For rectal cancer patients with a preoperative clinical stage of cT_3_/cT_4_ or N_+_, we recommend radical rectal resection. If postoperative pathology confirms negative surgical margins, it is defined as an R0 resection. Adjuvant concurrent chemoradiotherapy is advised, with postoperative chemotherapy options including single-agent chemotherapy or combination regimens. For metastatic rectal cancer with isolated, resectable liver metastases, radical rectal resection combined with liver metastasectomy is performed. If postoperative pathology confirms negative surgical margins, it is defined as an R0 resection. Postoperative chemotherapy regimens include single-agent chemotherapy or combination regimens such as CAPEOX (XELOX) and mFOLFOX6. For metastatic rectal cancer assessed as unresectable but accompanied by acute symptoms such as bleeding, perforation, or obstruction, palliative surgery is performed. These cases are defined as non-R0 resections, and postoperative chemoradiotherapy is recommended.

### 2.4 Data collection and follow-up

The basic information and the clinicopathological data were collected from the prospective cancer database in our institution. Basic information included the patient’s name, ID, gender, age, tobacco use, alcohol consumption, and family history of cancer, while the clinicopathological data contains tumor location, R0 resection, tumor differentiation, TNM stage, operative method, surgical duration, intraoperative blood loss, postoperative chemoradiotherapy, preoperative CEA, and preoperative TTR. Tobacco use was categorized into three levels: non-smoker, former smoker, and smoker. Alcohol consumption was similarly classified into three levels: non-drinker, light-to-moderate drinker (daily alcohol intake less than 2 units or occasional drinkers), and heavy drinker (daily alcohol intake greater than 2 units). Tumor location was further specified as proximal colon (from cecum to colon splenic flexure), distal colon (from descending colon to sigmoid colon), and rectum (from rectal junction to rectum) according to the International Classification of Diseases for Oncology. The pathology of each tissue sample was independently assessed by two experienced pathologists who were blinded to the TTR status. The Eighth Edition of the American Joint Committee on Cancer (AJCC) Staging for colorectal cancer was applied to determine the TNM stage system.

The follow-up protocol for all patients at our institution included regular assessments, involving telephone or outpatient reviews every 3 months during the initial 2 years post-surgery and subsequent evaluations every 6 months. Overall survival (OS) was characterized as the duration from the surgery date to the date of death, whereas cancer-specific survival (CSS) was specified as the duration from the surgery date to the date of death from cancer. The final follow-up date for the training cohort in this study was March 31, 2020, while for the validation cohort, it extended until December 31, 2021.

### 2.5 Statistical analysis

All analyses were performed with IBM SPSS Statistics 22.0 software, R version 4.3.1, and the X-tile program. Differences in two-tailed P values <0.05 were considered statistically significant. The X-tile software was utilized to estimate the optimal cutoff values for TTR (30). Continuous variables were presented as medians with interquartile ranges, while categorical variables were expressed as absolute numbers or percentages. Categorical variable comparisons were performed using the χ2 test or Fisher’s exact test, ordinal data were analyzed with the Kruskal-Wallis test, and continuous variables were compared using the Mann-Whitney U test. Kaplan-Meier method was used to calculate the survival rate and draw the survival curve, with the Log-Rank test utilized for survival analysis. The risk factors for CSS were determined by Univariate and multivariate Cox proportional hazards regression analysis and expressed using hazard ratio (HR) with a 95% confidence interval (CI). Based on risk factors identified through multivariate Cox analysis, several postoperative CSS prediction nomograms were developed. The nomograms underwent internal validation using the bootstrap method. External validation was performed by validating 378 patients in the validation cohort to evaluate the performance of the nomograms. The receiver operating characteristic (ROC) curve, calibration curve, concordance index (C-index), and decision curve analysis (DCA) were used to evaluate the discrimination ability and prediction effect of nomograms (31).

## 3 Results

### 3.1 Cut-off value of TTR

**Figure 2:**
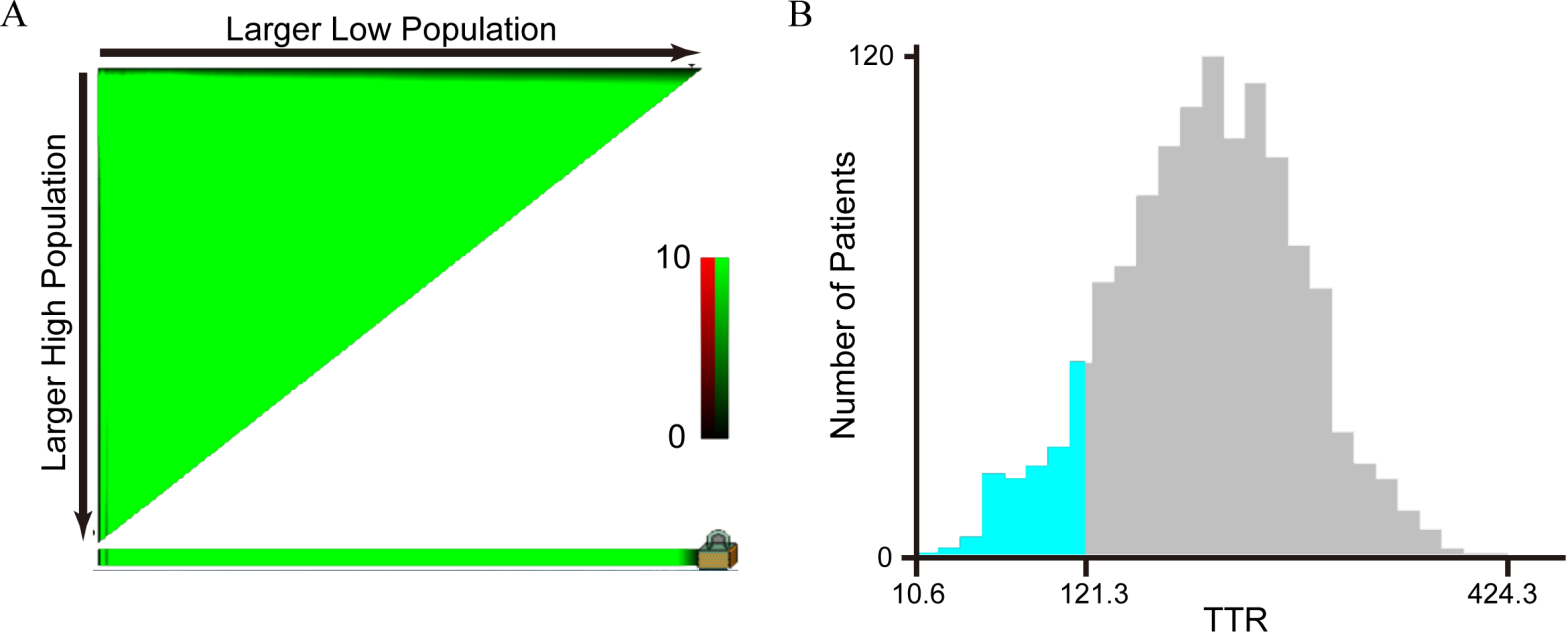
Analysis of the optimal cut-off value of TTR by X-tile software. **(A)** An X-tile plot of TTR; **(B)** The optimal cut-off value was highlighted by a histogram. The optimal cut-off value for TTR was 121.3 mg/L.

We used two methods to determine the optimal cut-off value for TTR classification. The first method involved grouping patients based on the lower normal value of 100 mg/L found in the literature, dividing the 1,322 colorectal cancer (CRC) patients into Group A (TTR < 100 mg/L) and Group B (TTR ≥ 100 mg/L). The second method used the X-tile software to determine the optimal cut-off value, categorizing the 1,322 CRC patients into Group C (TTR < 121.3 mg/L) and Group D (TTR ≥ 121.3 mg/L). The relationship between these two grouping methods and the cancer-specific survival is shown in **Table 1**. The results indicated that the hazard ratio (HR) for the second grouping method was 0.31(0.24-0.40), while the HR for the first grouping method was 0.35(0.25-0.48). Clearly, the most appropriate cut-off value of TTR for predicting cancer-specific survival (CSS) in CRC patients was determined to be 121.3 mg/L, as established by the X-tile software in the training cohort (**Figure 2**). Consequently, the 1,322 consecutive CRC patients were stratified into two groups: the TTR-lower group (TTR < 121.3 mg/L) and the TTR-higher group (TTR ≥ 121.3 mg/L).

**Table 1:** Two methods to determine the optimal cut-off value for TTR classification.

Characteristics		Number (%)	HR (95% CI)	P Value
Method 1	Group A (TTR<100mg/L)	82 (6.20%)	0.35 (0.25-0.48)	<0.001
	Group B (TTR ≥100mg/L)	1240 (93.80%)		
Method 2	Group C (TTR<121.3mg/L)	133 (10.1%)	0.31 (0.24-0.40)	<0.001
	Group D (TTR ≥121.3mg/L)	1189 (89.9%)		

### 3.2 Transthyretin and clinicopathological features

**Table 2** provides a comprehensive overview of the characteristics of all included patients and shows the division of the 1322 CRC patients. 133 patients (10.1%) were classified into the TTR-lower group, while 1189 (89.9%) were assigned to the TTR-higher group. In the TTR-lower group, the median age of the CRC patients was 66.0 years, which was older than the median age of 62.0 years observed in the TTR-higher group (p=0.004). Similar trends were observed in preoperative CEA levels, with the median CEA in the TTR-lower group being 4.74μg/L, significantly higher than the 3.39μg/L in the TTR-higher group (p=0.022). Furthermore, TTR was significantly associated with gender (p=0.032), location (p<0.001), R0 resection (p<0.001), differentiation (p=0.004) and stage (p<0.001). However, no significant differences were observed in tobacco use, alcohol consumption, family history of cancer, operative method, surgical duration, intraoperative blood loss and postoperative chemoradiotherapy (p>0.05). The basic characteristics of the validation cohort are shown in **Supplementary Table 1**. Additionally, we collected liver function parameters of the 1,322 CRC patients, as shown in **Table 3**. The results indicated that the TTR-lower group had slightly lower ALT and slightly higher GLOB levels compared to the TTR-higher group. However, the quartiles and medians of liver function parameters for both groups remained within the clinically normal range, suggesting that patients in the TTR-lower group did not exhibit significant liver dysfunction or impaired hepatic secretory function.

**Table 2:** Characteristics and clinicopathologic features of 1322 colorectal patients according to preoperative transthyretin levels.

Variables	Total Patients	TTR(mg/L)		p
		Lower (<121.3)	Higher (≥121.3)	
	N=1322	N=133	N=1189	
Age, [M (Q1,Q3),y]	62 (54.00,71.00)	66 (58,73)	62 (54,70)	0.004
Gender				
Male	761 (57.6)	65 (48.9)	696 (58.5)	0.032
Female	561 (42.4)	68 (51.1)	493 (41.5)	
Tobacco Use				
Non-smoker	925 (70.0)	94 (70.7)	831 (69.9)	0.358
Former Smoker	62 (4.7)	3 (2.3)	59 (5.0)	
Smoker	335 (25.3)	36 (27.1)	299 (25.1)	
Alcohol Consumption				
Non-drinker	1201 (90.8)	122 (91.7)	1079 (90.7)	0.928
Light-to-moderate Drinker	64 (4.8)	6 (4.5)	58 (4.9)	
Heavy Drinker	57 (4.3)	5 (3.8)	52 (4.4)	
Family History of Cancer				
No	1191 (90.1)	120 (90.2)	1071 (90.1)	0.858
Colorectal Cancer	28 (2.1)	2 (1.5)	26 (2.2)	
Other Cancers	103 (7.8)	11 (8.3)	92 (7.7)	
Location				
Proximal Colon	307 (23.2)	57 (42.9)	250 (21.0)	<0.001
Distal Colon	269 (20.3)	32 (24.1)	237 (19.9)	
Rectum	746 (56.4)	44 (33.1)	702 (59.0)	
R0 Resection				
No	52 (3.9)	16 (12.0)	36 (3.0)	<0.001
Yes	1270 (96.1)	117 (88.0)	1153 (97.0)	
Differentiation				
Low	168 (12.7)	28 (21.1)	140 (11.8)	0.004
Moderate	1085 (82.1)	102 (76.7)	983 (82.7)	
High	69 (5.2)	3 (2.3)	66 (5.6)	
Stage				
I	171 (12.9)	6 (4.5)	165 (13.9)	<0.001
II	591 (44.7)	62 (46.6)	529 (44.5)	
III	426 (32.2)	30 (22.6)	395 (33.3)	
IV	134 (10.1)	35 (26.3)	99 (8.3)	
IV (with liver metastases)	108 (8.2)	29 (21.8)	79 (6.7)	<0.001
Operative Method				
Laparotomy	282 (21.3)	31 (23.3)	251 (21.1)	0.557
Laparoscopic or robotic surgery	1040 (78.7)	102 (76.7)	938 (78.9)	
Surgical Duration				
<2 h	631 (47.7)	62 (46.6)	569 (47.9)	0.786
≥. h	691 (52.3)	71 (53.4)	620 (52.1)	
Intraoperative Blood Loss				
<50 ml	671 (50.8)	73 (54.9)	598 (50.3)	0.315
≥.0 ml	651 (49.2)	60 (45.1)	591 (49.7)	
Postoperative Chemoradiotherapy				
No	628 (47.5)	65 (48.9)	563 (47.4)	0.739
Yes	694 (52.5)	68 (51.1)	626 (52.6)	
Preoperative CEA, [M (Q1,Q3), μg/L]	3.52 (1.89,9.11)	4.74 (2.23,14.63)	3.39 (1.87,8.66)	0.022

**Table 3:** Liver function parameters of 1322 colorectal patients according to preoperative transthyretin levels.

Variables	Total Patients	TTR(mg/L)		p
		Lower (<121.3)	Higher (≥121.3)	
	N=1322	N=133	N=1189	
AST [M (Q1,Q3), (U/L)]	17.5 (14.3,21.9)	17.0 (13.0,23.5)	17.6 (14.6,21.7)	0.869
ALT [M (Q1,Q3), (U/L)]	13.9 (10.0,20.0)	10.9 (7.0,21.7)	14.0 (10.0,20.0)	0.019
ALP [M (Q1,Q3), (U/L)]	77.0 (65.2,92.0)	74.0 (61.5,93.7)	77.0 (65.4,92.0)	0.333
γ-GT [M (Q1,Q3), (U/L)]	16.6 (12.0,25.1)	17.9 (13.0,32.0)	16.3 (12.0,25.0)	0.129
DBil [M (Q1,Q3), (μmol/L)]	3.3 (2.3,4.5)	3.2 (2.2,4.4)	3.3 (2.4,4.5)	0.649
IDBil [M (Q1,Q3), (μmol/L)]	6.7 (4.9,9.4)	6.3 (4.7,7.7)	6.9 (5.1,9.5)	0.105
ALB [M (Q1,Q3), (g/L)]	38.5 (35.9,41.4)	36.6 (35.1,38.4)	39.0 (36.4,41.6)	0.093
GLOB [M (Q1,Q3), (g/L)]	25.9 (23.5,28.7)	27.8 (23.8,31.7)	25.8 (23.5,28.3)	<0.001

### 3.3 Associations between transthyretin level and patient survival

**Figure 3:**
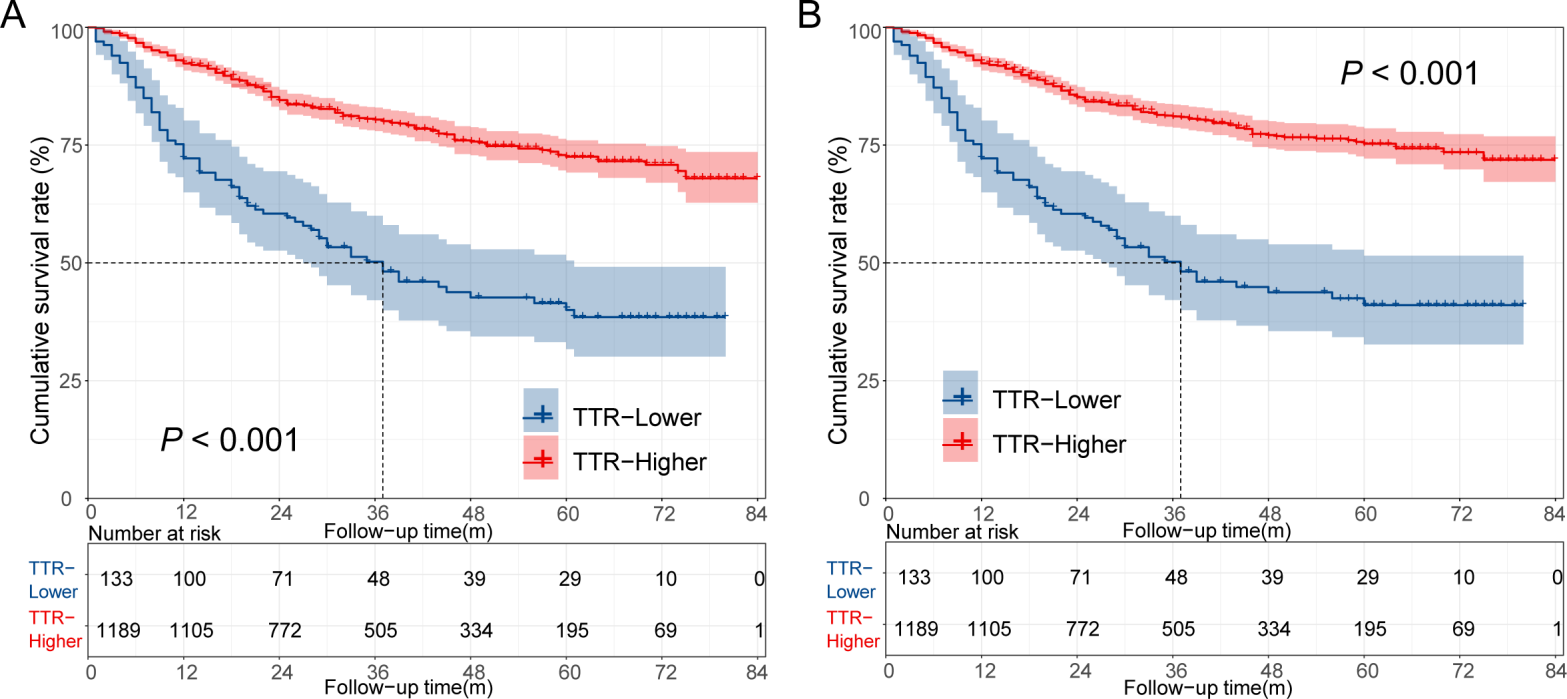
Overall survival curve **(A)** and cancer-specific survival curve **(B)** for different TTR levels in CRC patients underwent surgical treatment.

By the end of the follow-up period, a total of 317 deaths were observed in 1322 patients, including 298 cancer-specific deaths. The cumulative 1-year, 3-year, and 5-year of colorectal cancer-specific survival (CSS) rates in the TTR-lower group were 72.2%, 50.2%, and 41.0%, respectively. These were significantly shorter than the corresponding rates in the TTR-higher group, which were 92.3%, 81.1%, and 75.3%, respectively (p < 0.001 for all comparisons). Similar trends were observed in overall survival (OS), with the cumulative 1-year, 3-year, and 5-year OS rates in the TTR-lower group and TTR-higher group being 72.2% vs 92.3%, 50.2% vs 80.4%, and 40.0% vs 72.5% (p < 0.001 for all comparisons). The Kaplan-Meier survival estimate also indicated that the TTR-low group exhibited significantly worse OS and CSS than the TTR-high group (**Figure 3**).

**Figure 4:**
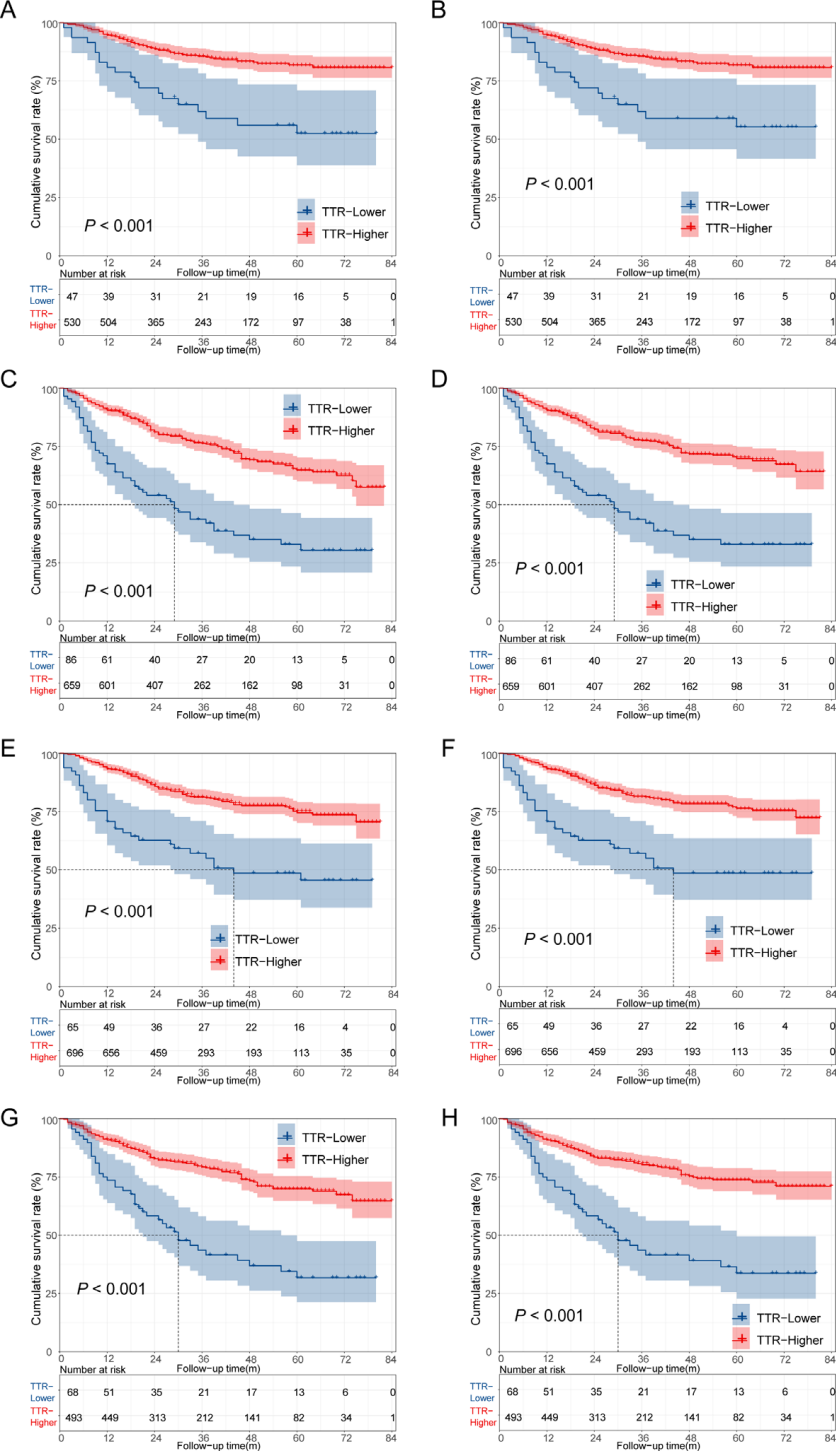
Kaplan–Meier survival analysis of colorectal cancer patients who underwent surgery. **(A)** The overall survival of CRC patients aged ≤60 years; **(B)** The cancer-specific survival of CRC patients aged ≤60 years; **(C)** The overall survival of CRC patients aged >60 years; **(D)** The cancer-specific survival of CRC patients aged >60 years; **(E)** The overall survival of male CRC patients; **(F)** The cancer-specific survival of male CRC patients; **(G)** The overall survival of female CRC patients; **(H)** The cancer-specific survival of female CRC patients.

Additionally, we conducted a stratified analysis based on the age and gender of the patients, dividing them into several subgroups. In patients aged ≤60 years, the TTR-higher group exhibited a more favorable prognosis compared to the TTR-lower group, with both the 5-year overall survival (OS) and cancer-specific survival (CSS) rates being higher in the TTR-high group than in the TTR-low group. (81.8%vs 52.5%, 81.8% vs 55.3%, p < 0.001 for both). Comparable conclusions were observed within the subgroups aged >60 years, male, and female, indicating a consistent trend across these demographic subsets (**Figure 4**).

### 3.4 Prognostic factors analysis for postoperative colorectal cancer-specific mortality

**Figure 5:**
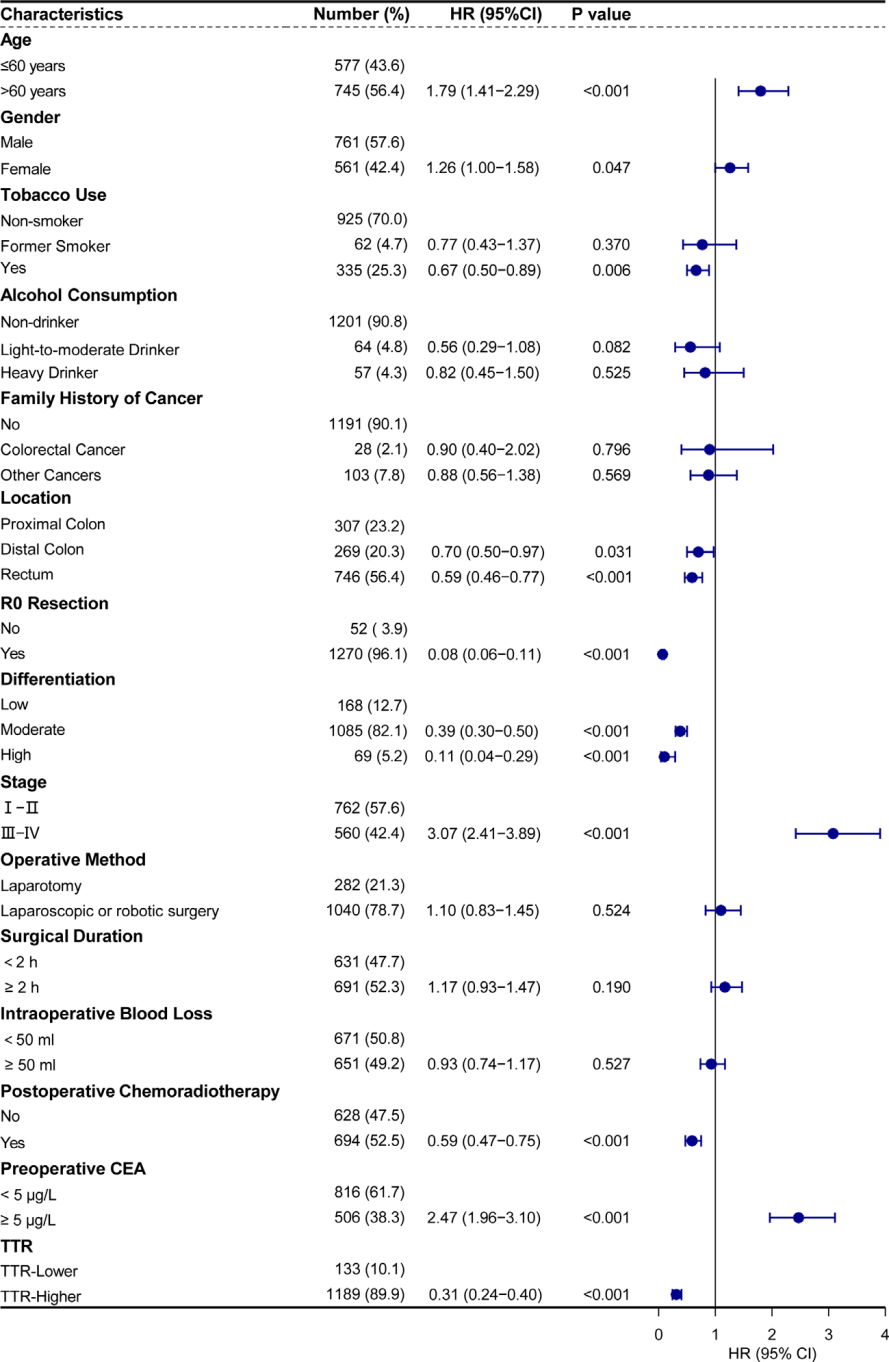
Univariate analysis of cancer-specific survival showed that TTR and other clinicopathological parameters were significant prognostic factors.

To identify the most appropriate variables for constructing a postoperative survival predictive model, we utilized a Cox proportional hazards regression model. In the univariate Cox regression analyses, age, gender, tobacco use, location, R0 resection, differentiation, stage, postoperative chemoradiotherapy, preoperative CEA, and TTR were identified as factors significantly affecting the prognosis of colorectal cancer patients undergoing surgery (all p <0.05) (**Figure 5**).

**Figure 6:**
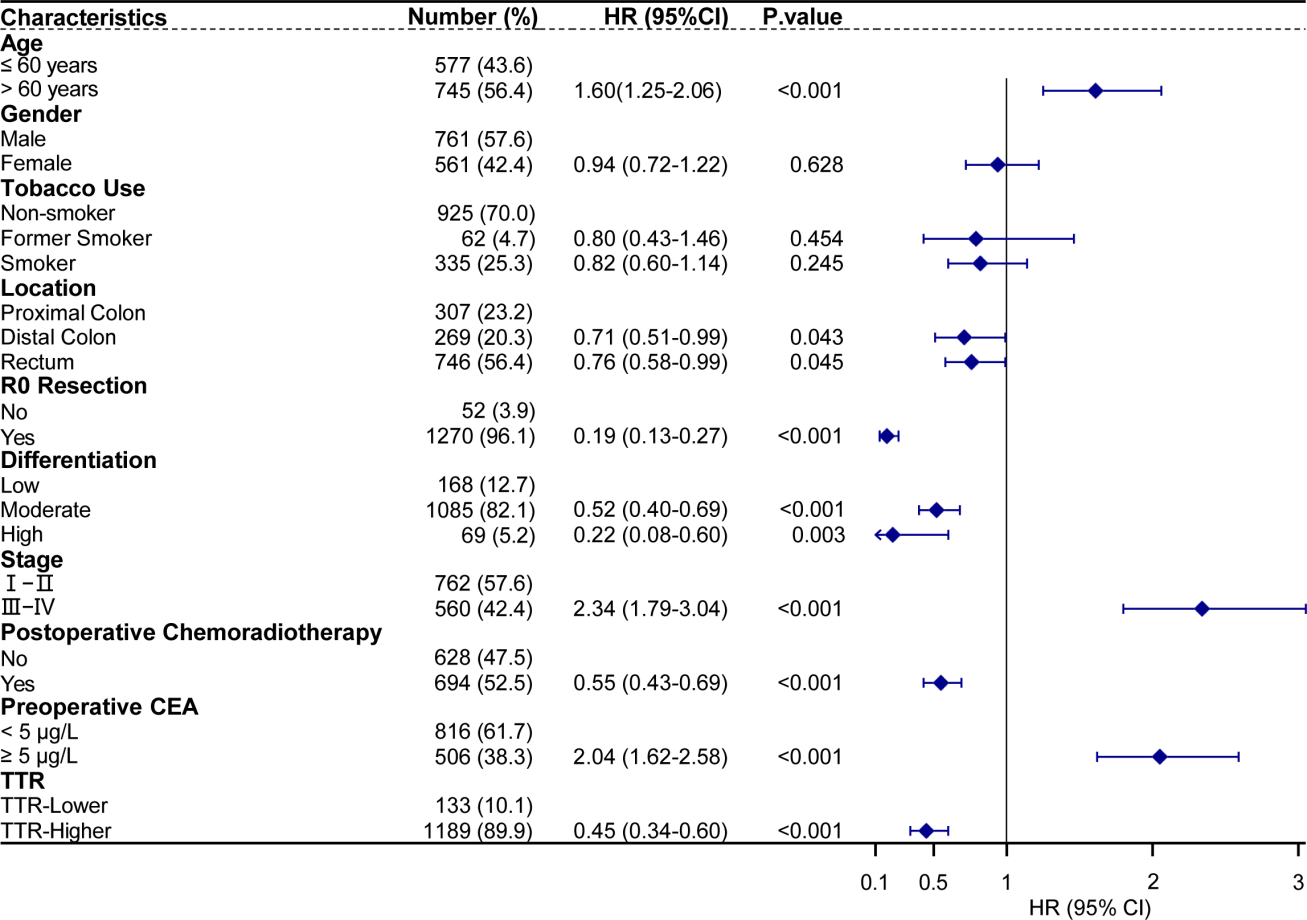
Multivariate analysis of prognostic factors with cancer-specific survival in 1322 CRC patients underwent surgical treatment.

In further multivariate Cox regression analyses, age>60 years (hazard ratio [HR] = 1.60, 95% confidence interval [CI] : 1.25–2.06, p<0.001), stage III-IV(HR = 2.34, 95% CI: 1.79–3.04, p<0.001), preoperative CEA≥5 (HR  = 2.04, 95% CI: 1.62–2.58, p<0.001) were identified as risk factors for postoperative cancer-specific mortality. Conversely, tumor located in the distal colon (HR = 0.71, 95% CI: 0.51–0.99, p = 0.043), located in the rectum (HR = 0.76, 95% CI: 0.58–0.99, p = 0.045), R0 resection (HR = 0.19, 95% CI: 0.13–0.27, p < 0.001), moderate differentiation (HR = 0.52, 95% CI: 0.40–0.69, p < 0.001), high differentiation (HR = 0.22, 95% CI: 0.08–0.60, p = 0.003), postoperative chemoradiotherapy (HR = 0.55, 95% CI: 0.43–0.69, p < 0.001), and higher TTR (HR = 0.45, 95% CI: 0.34–0.60, p < 0.001) were identified as protective factors for postoperative cancer-specific mortality (**Figure 6**).

### 3.5 Construction and internal validation of cancer-specific mortality prediction models

**Figure 7:**
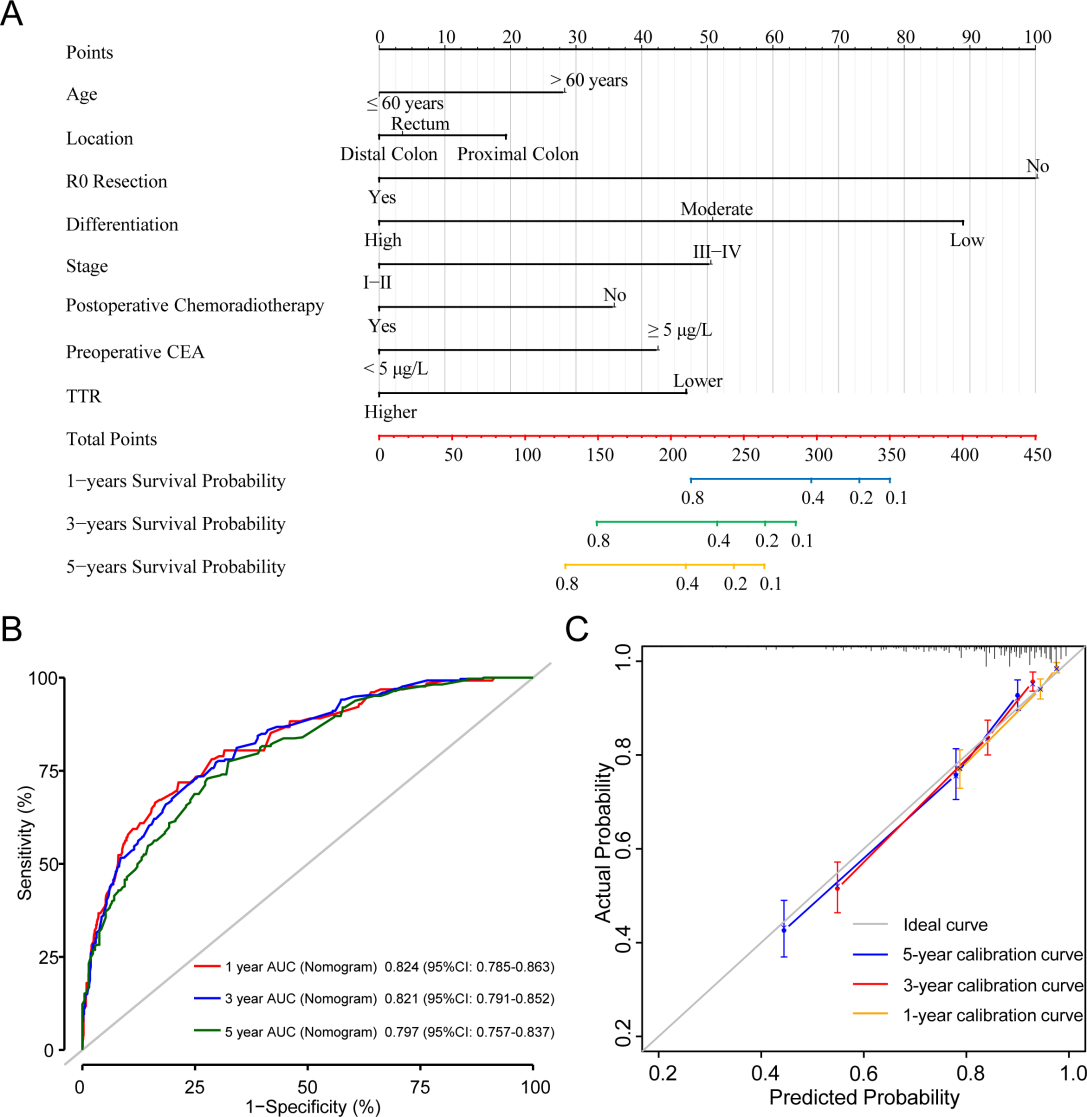
A Cox proportional hazards Model A of cancer specific mortality for CRC patients underwent surgical treatment. **(A)** The nomogram of Model A. **(B)** 1-, 3-, and 5-year ROC comparison of Model A. **(C)** 1-, 3-, and 5-year calibration curves of Model A.

We established Model A based on multivariate Cox regression analysis and accordingly could predict the 1-year, 3-year, and 5-year CSS of CRC patients (**Figure 7A**). The 1-year, 3-year and 5-year AUC predicted by Model A were 0.824 (95%CI: 0.785-0.863), 0.821 (95%CI: 0.791-0.852) and 0.797 (95%CI: 0.757-0.837) respectively, which exhibited its robust predictive performance. The C-index for Model A was 0.795 (95%CI: 0.769-0.821), underscoring its excellence as a classifier with high accuracy, particularly in short-term postoperative survival prediction (**Figure 7B**). Additionally, calibration curves for 1-year, 3-year, and 5-year intervals demonstrated the model’s strong calibration and discrimination capabilities (**Figure 7C**). Compared to the TNM staging used by the most common AJCC in clinical practice, model A consistently displayed higher AUC values at any given time point within the first 5 years post-surgery (**Supplementary Figure 1**).

### 3.6 Comparison and external validation of mortality prediction models

**Figure 8:**
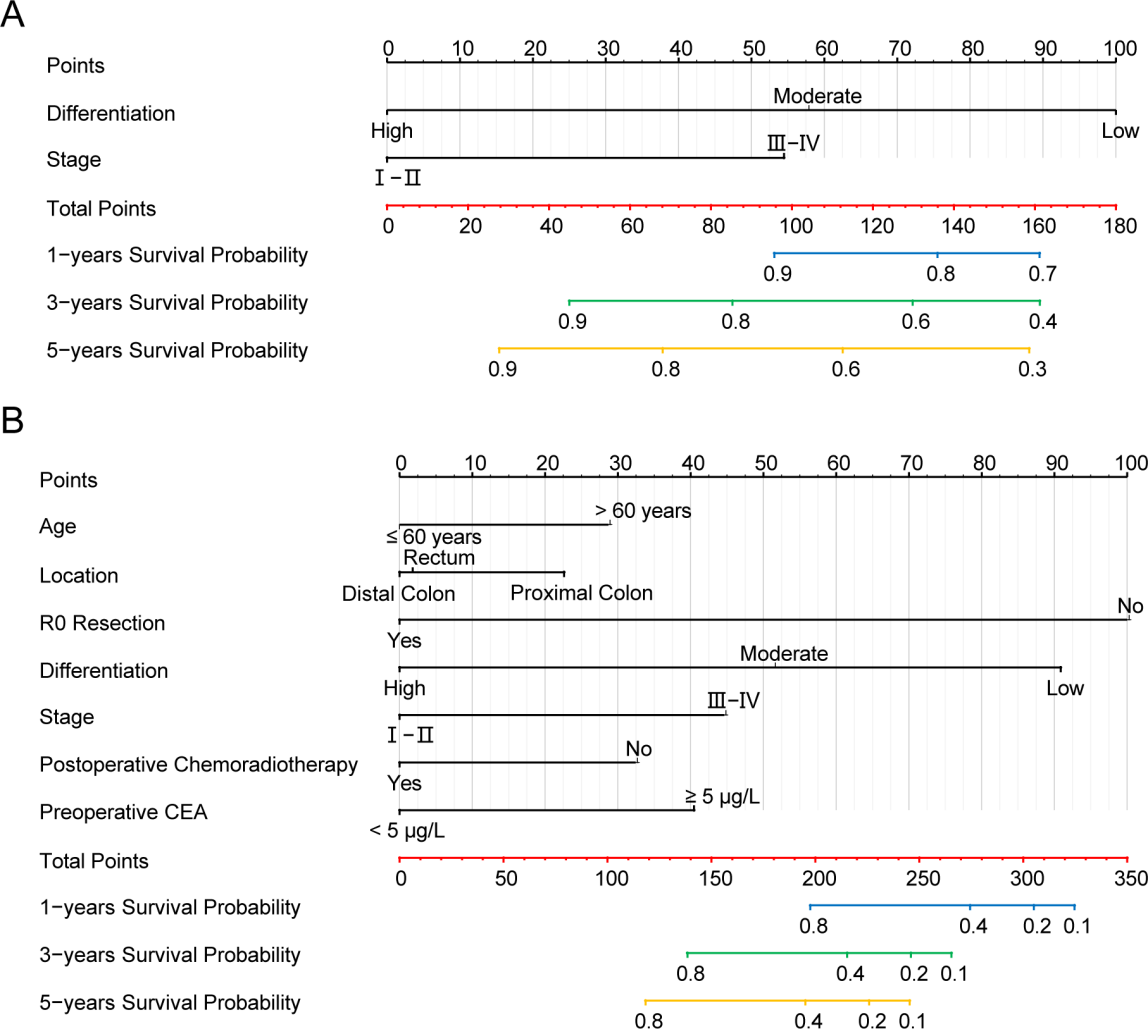
**(A)** Nomogram Model B and **(B)** Nomogram Model C.

In addition to Model A, we introduced Model B and Model C into our analysis. Model B incorporated all parameters that were significant in the multivariate Cox regression analysis without preoperative TTR level, while Model C was established only containing the TNM stage and tumor differentiation (**Figure 8**). The 1-year,3-year, and 5-year ROC of the three models are shown in **Supplementary Figure 2**, revealing that Model A exhibited the highest AUC. Additionally, the time-ROC curve analysis of the three models indicated that the AUC of Model A for predicting postoperative CSS in colorectal cancer is the highest across all periods (**Supplementary Figure 3**). The DCA curve shows that compared to Model B and Model C, the most effective decision-making results can be obtained when utilizing the Model A to determine the prognosis of colorectal cancer patients undergoing surgery (**Supplementary Figure 4**). In the external cohort validation comprising 377 individuals, ROC curves, calibration plots, and Decision Curve Analysis (DCA) plots consistently revealed similar results, emphasizing the distinctive superiority of Model A in predicting the prognosis of colorectal cancer patients undergoing surgery (**Supplementary Figures 5**–**7**).

## 4 Discussion

Colorectal cancer constitutes a global public health challenge that demands comprehensive solutions. This malignancy not only poses a severe threat to human health but also imposes significant burdens on families and society at large. The considerable variability in prognosis and survival among patients with the same cancer type is attributed to the diverse biological behaviors of tumors. The identification of meaningful prognostic factors and the development of predictive models serve multifaceted purposes. They not only aid patients in formulating personalized treatment strategies based on scientific evidence but also play a pivotal role in preventing overtreatment and conserving valuable medical resources. Furthermore, these efforts assist patients in planning their remaining lives and contribute to enhancing their sense of life dignity.

In this clinical study, we explored the relationship between TTR and clinical outcomes in colorectal patients undergoing surgery through a training cohort of 1322 patients. In the process of patient recruitment, we excluded patients over 80 years old for the consideration of cardiovascular accidents, stroke, depression, lung disease, and other factors that can affect OS, and we were more concerned about the impact of TTR on CSS. The findings indicated a correlation between reduced TTR levels and unfavorable postoperative prognosis, proposing TTR as a potential marker for predicting adverse outcomes in colorectal cancer surgery patients. These results align with studies on other digestive tract tumors like gastric and liver cancers (26, 27). We then conducted Cox regression analysis on the training cohort to identify risk factors for CSS of CRC. Subsequently, based on the results of multivariate Cox analysis, we developed a prognostic nomogram. Additionally, several nomograms were constructed based on various clinical-pathological information. The outcomes of internal validation using the bootstrap method demonstrated a strong concordance between predicted and observed results, which was supported by robust ROC curve analysis, a C-index exceeding 0.75, and a well-fitted calibration curve, indicating substantial discriminatory ability. Furthermore, the external validation was also conducted in the validation cohort of 377 patients, which indicates the high accuracy of CSS predictions for 1-, 3- and 5-year periods. The DCA demonstrated that the nomogram we developed was superior to other models, which means the nomogram possesses substantial clinical practical value (32). This emphasizes the enhanced prognostic accuracy achieved through the incorporation of TTR in the predictive model.

Malignant tumors are recognized as chronic, consumptive diseases, particularly within the digestive system, exerting a substantial and notable impact on nutrient depletion and weight loss. The stress and trauma associated with major surgical procedures exacerbate nutrient catabolism, leading to inadequate nutritional reserves and slower postoperative recovery. Some studies indicate that malnutrition can impact the postoperative recovery of surgical patients, leading to unfavorable outcomes (33). Therefore, preoperative nutritional markers deserve attention and should not be overlooked as they play a crucial role in evaluating the prognostic status of patients. Many nutritional indicators have been considered for clinical practice, including BMI, albumin, and prognostic nutritional index (PNI) (34). However, different nutritional indicators have their scope of application and limitations. BMI focuses on weight, but it ignores factors that affect one person’s health, including gender, age, and ethnicity (35). In other literature, a series of indicators based on albumin have been used to predict the prognosis of cancer, including PNI and nutrition risk index. Some researchers insisted that they may be useful indicators in the prognosis of digestive system neoplasms (36, 37). However, other scholars have argued that the prognostic significance of indicators based on albumin was not significant in patients with early-stage gastric cancer (38).

TTR is nowadays often preferred over albumin, given its shorter half-life of 2 days, significantly shorter than the 3 weeks of albumin (39). This characteristic allows TTR to capture more rapid changes in the nutritional state compared to other indicators. Furthermore, an epidemiological survey in Australia showed that TTR is not affected by racial differences or genetic factors, suggesting that it can be used as a more sensitive and reliable indicator for evaluating liver function and recent nutritional status than albumin or PNI (40). It has been widely confirmed that serial measurement of TTR allows monitoring of fluctuations in lean body mass (LBM) depletion and predicting the outcome of critically ill patients, including burns, polytrauma, septicemic, or neoplastic invasion (41, 42). Some research institutions in Europe and East Asia have suggested that TTR should be used as a routine nutritional monitoring indicator (24, 43–47). Therefore, TTR has been employed to predict the occurrence of surgical complications, differentiate between inflammation and malignancy, and predict the prognosis of patients diagnosed with malignant tumors. This underscores its potential utility as a marker in oncology.

The normal TTR concentration in adult males is stable at approximately 310 mg/L, while it is approximately 260 mg/L in adult females (39). Different researchers have chosen different TTR concentrations as the cut-off value in their studies. In a clinical study around non-small cell lung cancer, researchers chose 220 mg/L as the cutoff value for TTR, and patients below this level would have poorer disease-free survival (28). The researchers selected 200 mg/L as the optimal cut-off value based on the most prominent points on the ROC curves and verified that the preoperative TTR was a prognostic factor in patients with adenocarcinoma of the esophagogastric junction (48). The X-tile program identified 180 mg/L as the TTR cut-off value, revealing that TTR levels serve as a more sensitive index of nutritional change and superior indicators of prognosis compared to albumin levels in stage II/III gastric cancer (49). In a clinical trial studying hepatocellular carcinoma, researchers used a cut-off value of 170 mg/L for preoperative prealbumin level and confirmed that preoperative prealbumin level could be used in predicting long-term prognosis for patients undergoing liver resection (50). In adult subjects with other diseases, a threshold of 100 mg/L has been associated with ominous prognostic significance, potentially indicating the depletion of Lean Body Mass (LBM) resources (51, 52). The range of cut-off values from 200 mg/L to 100 mg/L delineates a gray zone within which TTR concentrations may fluctuate, indicating potential outcomes ranging from the best to the worst. The chosen cut-off value for TTR in our study was 121.3 mg/L, falling within the range of 100 mg/L to 200 mg/L, but lower than values ranging from 170 mg/L to 220 mg/L. The distinction in cut-off values can be attributed to the differential impact of colorectal cancer and gastric cancer on physiological functions. Colorectal cancer predominantly affects absorption function, resulting in symptoms such as diarrhea and loss of intestinal fluid. In contrast, gastric cancer primarily interferes with the stomach’s ability to grind food. Meanwhile, colorectal cancer might be detected at more advanced stages, leading to more severe nutritional deficiencies by the time of diagnosis compared to gastric cancer, which might be detected earlier. Despite slight variations in the cut-off value utilized within our study, the results consistently indicate a strong association between preoperative TTR and long-term prognosis.

While the exact reasons behind the robust connection between low TTR levels and the unfavorable prognosis of CRC patients remain unclear, potential mechanisms may be attributed to the following factors: Firstly, the initiation and progression of colorectal cancer are associated with an imbalance in the microecology of the digestive tract. Harmful strains such as Fusobacterium nucleatum and Bacteroides fragilis play a role in activating the NF-κB signaling pathway. This activation results in the secretion of cytokines, including tumor necrosis factor-alpha (TNF-α), interleukin-1 (IL-1), and IL-6, leading to inflammation which may further exacerbates the development of tumors (53). At the same time, these inflammatory factors are absorbed by the mucosa and enter the portal vein system into the liver, inhibiting the synthesis of prealbumin in an inflammatory state (54). Secondly, TTR carries retinoic acid by transporting retinol-binding protein. Research indicates that retinoic acid plays a crucial role in inhibiting the occurrence and development of colon cancer (55, 56). The decrease in TTR produced by the liver results in a reduction in the blood’s retinoic acid content, diminishing its inhibitory effect on cancer progression and potentially contributing to the rapid development of colorectal cancer. Furthermore, TTR has demonstrated potential roles in immune system regulation by influencing the differentiation of myeloid cells, thus modulating the tumor environment and controlling immune cell function (57).

There are several deficiencies and limitations in our clinical study. Above all, it is essential to acknowledge that this clinical study is a non-randomized, observational, and retrospective study, with patient selection limited to a single research center; the strict exclusion criteria resulted in a considerable loss of many patients, potentially introducing some selection bias. Secondly, it has become a consensus that patients with advanced colorectal cancer should undergo neoadjuvant chemoradiotherapy before surgery in recent years. However, the patients in this study came from several years ago, when the concept of neoadjuvant therapy was not widely accepted; meanwhile, although neoadjuvant chemoradiotherapy can benefit patients by reducing tumor staging, it inevitably causes side effects, such as damage to liver function and affecting food intake, which in turn affects preoperative TTR levels. Therefore, this study does not apply to the prognosis prediction of patients with colorectal cancer undergoing neoadjuvant therapy. Thirdly, owing to the limited number of patients, and the majority originating from the northwest region of China, there may be a homogeneity in the genotype of CRC patients, potentially influencing the prognosis. Whether the results in different periods, different regions, or different populations are consistent with the model has not been verified. Therefore, we expect a multicenter, large sample cohort to validate our results to confirm its prognostic role in colorectal cancer patients in the future.

## 5 Conclusion

Our study showed that a preoperative TTR < 121.3 mg/L emerged as a prognostic factor, predicting the long-term outcomes of CRC patients following surgical intervention. The predictive nomogram established on the basis of TTR exhibited robust predictive efficacy, as confirmed through both internal and external validation, supporting its role as a prognostic biomarker in clinical practice and offering valuable guidance for patient management.

## Data Availability

The original contributions presented in the study are included in the article/**Supplementary Material**. Further inquiries can be directed to the corresponding author.
