# Supplementary material for: Low transthyretin is associated with the poor prognosis of colorectal cancer
Source: Front Oncol. 2025 Feb 5;15:1397019. doi: 10.3389/fonc.2025.1397019 (PMC11835676; doi:10.3389/fonc.2025.1397019)
Supplement: Supplementary file 1 [file DataSheet1.docx]

**Low Transthyretin is Associated with the Poor Prognosis of Colorectal Cancer**

Zhang et al.

(Supplementary Figures)

**Supplementary Table. 1** Demographic and perioperative characteristics of external validation cohort (n=377)

| Parameters | |  | Results |
| --- | --- | --- | --- |
| Age, y |  | | 62.0 (52.5, 70.5) |
| Gender |  | |  |
|  | Male | | 215 (57.0) |
|  | Female | | 162 (43.0) |
| Tobacco Use |  | |  |
|  | Non-smoker | | 260 (69.0) |
|  | Former Smoker | | 25 (6.6) |
|  | Smoker | | 92(24.4) |
| Alcohol Consumption |  | |  |
|  | Non-drinker | | 337 (89.4) |
|  | Light-to-moderate Drinker | | 25 (6.6) |
|  | Heavy Drinker | | 15 (4.0) |
| Family History of Cancer |  | |  |
|  | No | | 338 (89.7) |
|  | Colorectal Cancer | | 10 (2.7) |
|  | Other Cancers | | 29 (7.7) |
| Location |  | |  |
|  | Proximal Colon | | 89 (23.6) |
|  | Distal Colon | | 77 (20.4) |
|  | Rectum | | 211(56.0) |
| R0 Resection |  | |  |
|  | No | | 21 (5.6) |
|  | Yes | | 356 (94.4) |
| Differentiation |  | |  |
|  | Low | | 48 (12.7) |
|  | Moderate | | 310 (82.2) |
|  | High | | 19(5.0) |
| Stage |  | |  |
|  | Ⅰ-Ⅱ | | 215 (57.0) |
|  | Ⅲ-Ⅳ | | 162 (43.0) |
| Operative Method |  | |  |
|  | Laparotomy | | 74 (19.6) |
|  | Laparoscopic or robotic surgery | | 303 (80.4) |
| Surgical Duration |  | |  |
|  | ＜2 h | | 158 (41.9) |
|  | ≥2 h | | 219 (58.1) |
| Intraoperative Blood Loss |  | |  |
|  | ＜50 ml | | 182 (48.3) |
|  | ≥50 ml | | 195 (51.7) |
| Postoperative Chemoradiotherapy | | |  |
|  | No | | 188 (49.9) |
|  | Yes | | 189 (50.1) |
| Preoperative CEA |  | |  |
|  | Normal | | 241 (63.9) |
|  | High | | 136 (36.1) |
| Preoperative TTR |  | |  |
|  | TTR-Low | | 58 (15.4) |
|  | TTR-High | | 319 (84.6) |
| CEA: Carcinoembryonic Antigen; TTR:Transthyretin | | | |


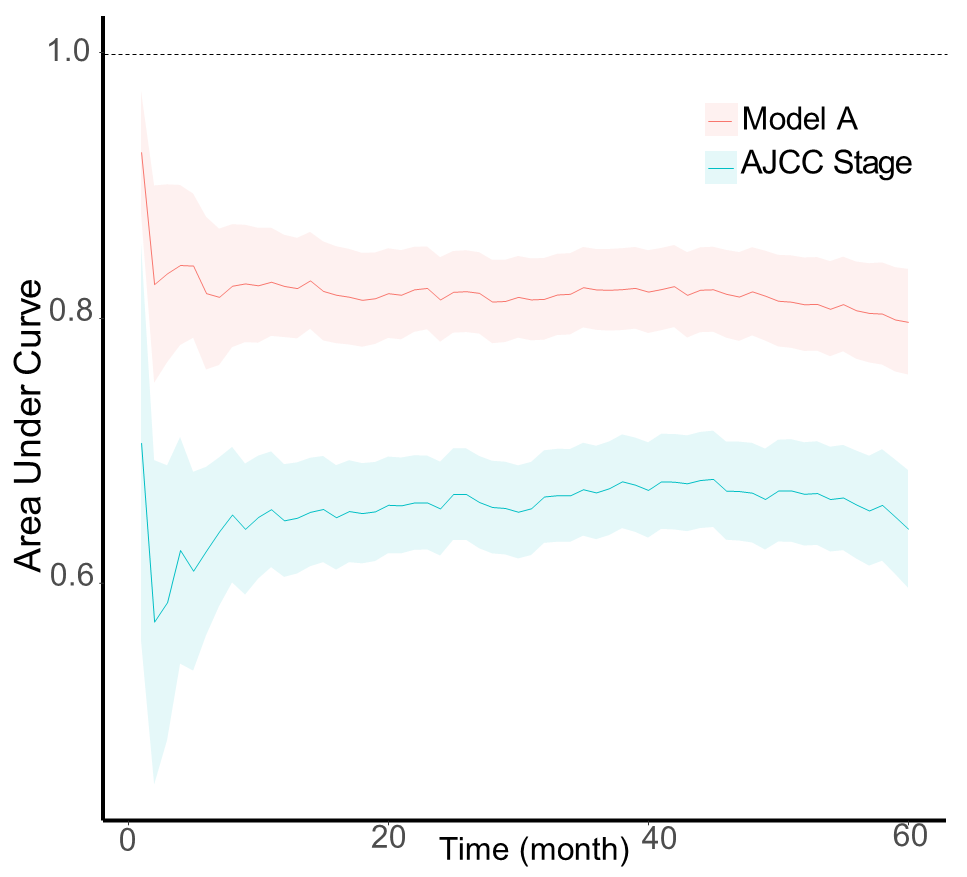


**Supplementary Figure. 1** Time-dependent ROC curves of Cox proportional hazards Model A and AJCC stage.


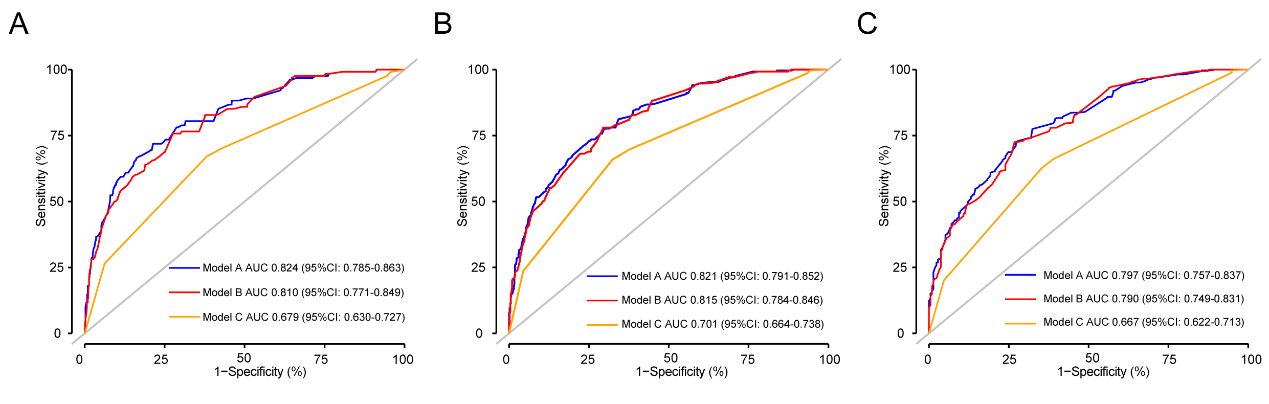


**Supplementary Figure. 2** ROC curves for different models in the prediction of the cancer-specific death of patients at 1- (A), 3- (B) and 5-year (C) point.


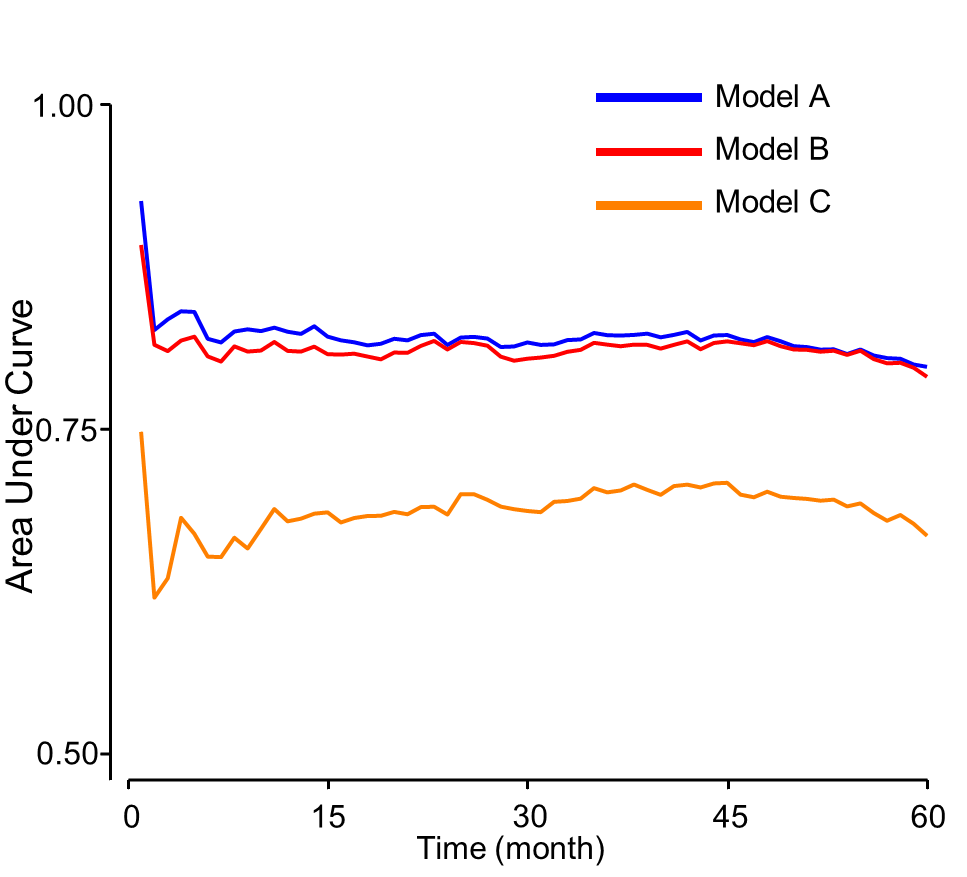


**Supplementary Figure. 3** The time-ROC curves of Model A , Model B and Model C


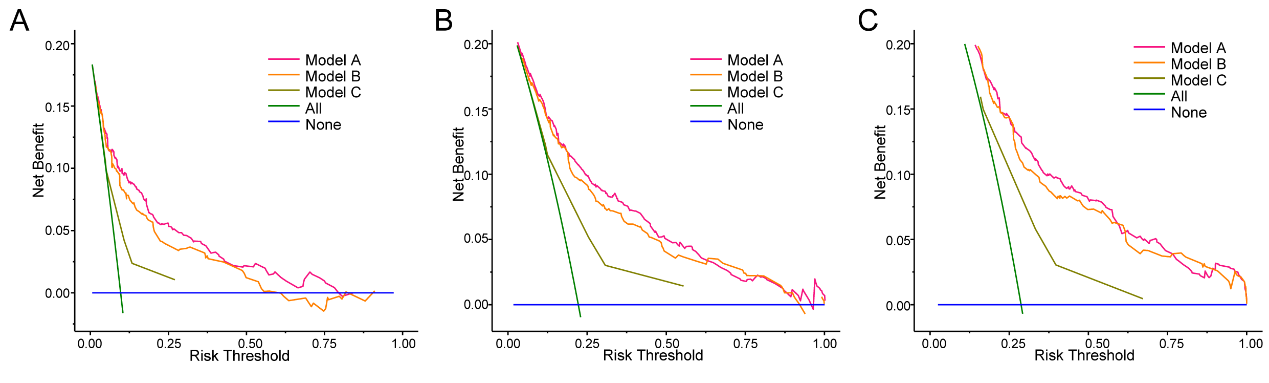


**Supplementary Figure. 4** Decision curve analysis for different models in the prediction of the cancer-specific death of patients 1- (A), 3- (B) and 5-year (C) point.


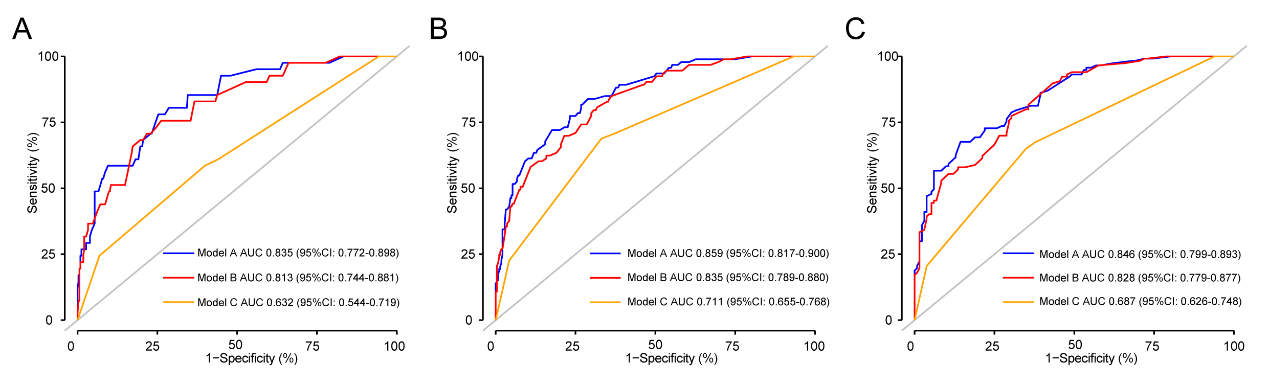


**Supplementary Figure. 5** ROC curves for different models in the prediction of the cancer-specific death of patients at 1- (A), 3- (B) and 5-year (C) point in external validation cohort.


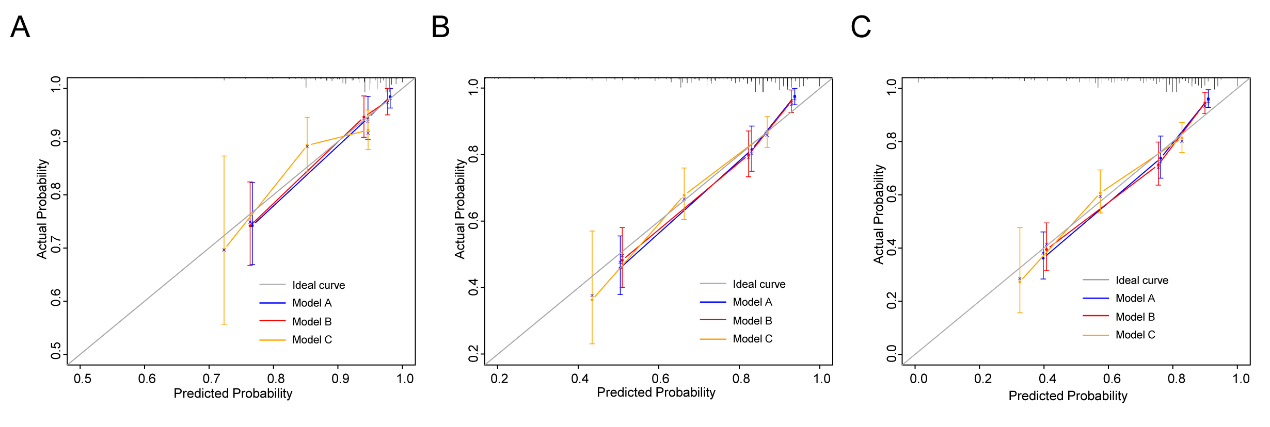


**Supplementary Figure. 6** Calibration plots for different models in the prediction of the cancer-specific death of patients at 1- (A), 3- (B) and 5-year (C) point in external validation cohort.


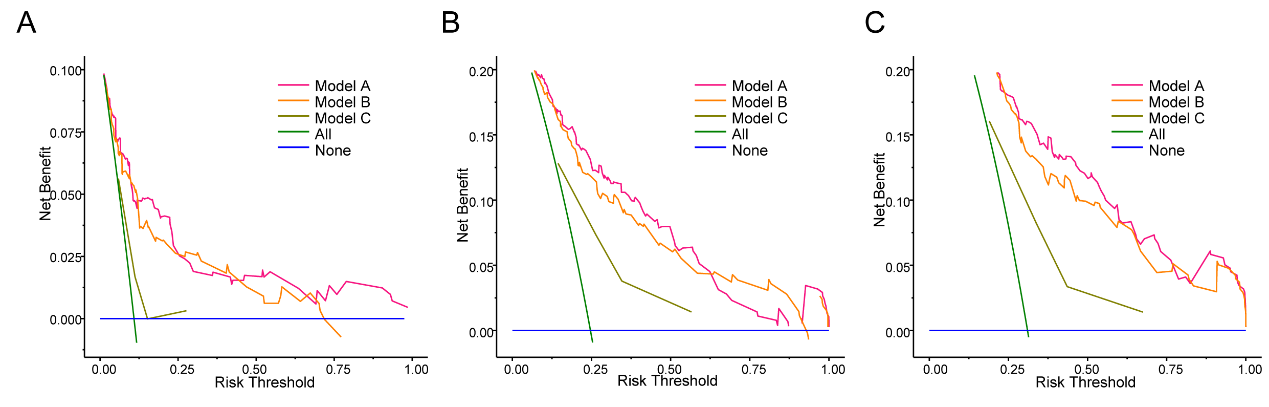


**Supplementary Figure. 7** Decision curve analysis for different models in the prediction of the cancer-specific death of patients at 1- (A), 3- (B) and 5-year (C) point in external validation cohort.
